# Substance P Receptor Antagonism: A Potential Novel Treatment Option for Viral-Myocarditis

**DOI:** 10.1155/2015/645153

**Published:** 2015-03-02

**Authors:** Prema Robinson, George E. Taffet, Nikita Engineer, Mitra Khumbatta, Bahrom Firozgary, Corey Reynolds, Thuy Pham, Tushar Bulsara, Gohar Firozgary

**Affiliations:** ^1^Department of Medicine, Baylor College of Medicine, 1 Baylor Plaza, Houston, TX 77030, USA; ^2^Department of Molecular Physiology and Biophysics, Baylor College of Medicine, Houston, TX 77030, USA

## Abstract

Viral-myocarditis is an important cause of heart failure for which no specific treatment is available. We previously showed the neuropeptide substance P (SP) is associated with the pathogenesis of murine myocarditis caused by encephalomyocarditis virus (EMCV). The current studies determined if pharmacological inhibition of SP-signaling via its high affinity receptor, NK1R and downstream G-protein, Ras homolog gene family, member-A (RhoA), will be beneficial in viral-myocarditis. Aprepitant (1.2 mg/kg), a SP-receptor antagonist, or fasudil (10 mg/kg), a RhoA inhibitor, or saline control was administered daily to mice orally for 3 days, prior to, or 5 days following, intraperitoneal infection with and without 50 PFU of EMCV, following which disease assessment studies, including echocardiogram and cardiac Doppler were performed in day 14 after infection. Pretreatment and posttreatment with aprepitant significantly reduced mortality, heart and cardiomyocyte size, and cardiac viral RNA levels (*P* < 0.05 all, ANOVA). Only aprepitant pretreatment improved heart functions; it significantly decreased end systolic diameter, improved fractional shortening, and increased peak aortic flow velocity (*P* < 0.05 all, ANOVA). Pre- or posttreatment with fasudil did not significantly impact disease manifestations. These findings indicate that SP contributes to cardiac-remodeling and dysfunction following ECMV infection via its high affinity receptor, but not through the Rho-A pathway. These studies suggest that SP-receptor antagonism may be a novel therapeutic-option for patients with viral-myocarditis.

## 1. Introduction

Viral-myocarditis is an important cause of heart failure among adolescents and young adults [1]. Myocarditis is caused most commonly in developed countries by viral infections such as coxsackie virus, echovirus, adenovirus, and picornavirus [2–5]. There is currently no specific treatment for viral-myocarditis [5]. Murine myocarditis caused by infection with encephalomyocarditis virus (EMCV) is a commonly used experimental model to study viral-myocarditis [6–9]. EMCV infection in mice is a fatal disease that leads to cardiac inflammation, dilated cardiomyopathy, and heart failure [6–9].

Substance P (SP) is a neuropeptide involved in pain transmission especially associated with inflammation [10–13]. Substance P is produced by neurons, endothelial cells, and immunocytes, such as lymphocytes and macrophages; receptors for SP are expressed on all these cells as well as on the surface of cardiomyocytes [14–17]. SP has been demonstrated by us and others to contribute to the pathogenesis of several viral, protozoan, and helminth infections in mouse and man [8, 18–20].

Rho is a member of the Ras superfamily of small GTP-binding proteins that play a central role in diverse biological processes such as actin cytoskeleton organization, microtubule dynamics, gene transcription, oncogenic transformation, cell cycle progression, adhesion, and epithelial wound repair [21, 22].

The SP receptor, neurokinin 1 receptor (NK1R), is a G-protein coupled receptor. Binding of SP to NK1R leads to activation of upstream regulators of RhoA activity [23, 24]. Signals elicited by G-protein coupled receptor activation have been shown to induce cardiomyocyte hypertrophy [25, 26]. RhoA activation is known to be involved in hypertrophy of neonatal rat ventricular myocytes [26–33]. Also mice overexpressing RhoA are known to develop a lethal dilated cardiomyopathy associated with heart failure [34, 35]. We have previously showed that SP is associated with the pathogenesis of EMCV infection in mice [8]. EMCV-infected wild type mice have 51% mortality, which was accompanied by increased cardiac SP protein, myocarditis, and cardiomyopathy [8] and increased viral levels (unpublished results). In contrast, SP−/− mice were completely protected from mortality following EMCV infection and demonstrated no pathology [8] and markedly reduced viral levels (unpublished results). SP is known to interact with 3 receptors, mainly NK1, but to a lesser extent, NK2 and NK3 [36]. There are no existing antagonists that inhibit all 3 receptors at the same time. Therefore although the SP knockout studies are illuminating, these studies cannot be directly extrapolated in humans. In order to test the effects of SP inhibition, individual SP-receptor antagonists need to be used. However, since NK1 is the predominant receptor that mediates SP responses in the heart [37], for the current studies, we used a NK1 receptor antagonist. We hence hypothesized that pharmacological inhibition of SP-signaling via its high affinity receptor, NK1R and/or downstream G-protein, Ras homolog gene family, member-A (RhoA), will be beneficial in viral-myocarditis.

## 2. Methods 

### 2.1. EMCV Model of Murine Myocarditis

This study was carried out in strict accordance with the recommendations in the Guide for the Care and Use of Laboratory Animals of the National Institutes of Health. The protocol was approved by the Committee on the Ethics of Animal Experiments of the Baylor College of Medicine (IACUC protocol number-AN209). All efforts were taken to ameliorate animal suffering. Mice were housed under BSL2 biohazard facility conditions. The mice were observed twice daily for the duration of the experiments. Mice that became moribund were considered to have reached the end point of the experiment and were humanely euthanized.

Male C57BL/6J mice that were used for the EMCV infection studies were purchased from The Jackson laboratory. The EMCV model of murine myocarditis used in these studies was as previously described [8]. Aprepitant (NK1R antagonist, Emend, Merck, Whitehouse station, NJ) was administered orally by gavage at a dosage of 1.2 mg/kg body weight. Fasudil (Rho-A inhibitor, Sigma, St Louis, MO) was administered orally by gavage at a dosage of 10 mg/kg. We administered the drugs daily, for 3 days (either prior to, or 5 days following, intraperitoneal infection with EMCV). We selected this dose of aprepitant since this dose is equivalent to that used by us in another murine infection and to treat chemotherapy-associated nausea in humans [19, 38]. The dose of fasudil is equivalent to the dose that was used by other investigators to mediate cardiovascular protection including significant reduction in infarct size following myocardial ischemia [39, 40]. Fifty plaque-forming units (pfu) of a myocarditic variant of EMCV that was generously provided by Dr. Sally Huber (University of Vermont) was injected intraperitoneally into 8-week-old male wild type mice. Controls included uninfected mice that were orally administered saline (sham), aprepitant, or fasudil alone. On day 14 post-infection, evidence for dilated cardiomyopathy or congestive heart disease was determined by performing echocardiograms and cardiac Dopplers to study functional changes such as changes in left ventricle systolic and diastolic diameter (ESD and EDD), fractional shortening, and peak aortic blood velocity. Functional studies were only performed on mice that survived to fourteen days which may have excluded those with the most cardiac dysfunction. Doppler studies and echocardiography studies were performed as outlined below [41, 42]  following which mice were humanely sacrificed and hearts harvested. Following were the manifestations studied: heart-to-body weight ratio, cardiac inflammatory score, cardiomyocyte diameter determination, and cardiac viral levels, as previously described [8]. Briefly, mice were weighed and anesthetized followed by sacrificing by cervical dislocation. The heart was harvested and weighed. The heart weight relative to body weight was calculated.

For determination of the inflammatory score, hearts were fixed in 4% paraformaldehyde, embedded in paraffin, stained with hematoxylin and eosin, and examined microscopically at 200x magnification. The inflammatory score was determined as described by Kanda et al. [43] and was as follows: 0, no lesions; 1+, lesions involving <25% of the ventricular myocardium; 2+, lesions involving 25–50% of the myocardium; 3+, lesions involving 50–75% of the myocardium; and 4+ lesions involving 75–100% of the myocardium.

Evidence of cardiomyocyte hypertrophy was determined by measuring diameter of cardiomyocytes following histopathological processing of the harvested hearts. Briefly, hearts were fixed in 4% paraformaldehyde, embedded in paraffin, sectioned, stained with hematoxylin and eosin, and examined microscopically at 200x magnification. Cardiac hypertrophy was determined by measuring mean cardiomyocyte diameter as determined by measuring the diameter of 50 myocytes using NIH IMAGE v.1.62 software.

Viral quantification was determined by real time PCR analysis using the SYBR Green Superscript One-Step Kit (Invitrogen, Carlsbad, CA) and 100 ng of RNA and specific primers: for EMCV (forward 5′-GTCGTGAAGGAAGCAGTTCC-3′; reverse 5′-CACGTGGCTTTTGGCCGCAGAGGC-3′; obtained from Sigma Genosys, St. Louis, MO, and for murine GADPH, forward, 5′-FTGTTCCTACCCCCAATGTGT-3′; reverse, 5′-CCTGCTTCACCACCTTCTTG-3′ from Invitrogen).

### 2.2. Echo Procedure

Using a Vevo 770 ultrasound machine equipped with a 30 MHz transducer (Visualsonics, Toronto, Canada),* in vivo* cardiac function and morphology were assessed. Mice were anesthetized in an induction chamber using 2.5% isoflurane and then transferred to a heated ECG platform for heart rate monitoring during the imaging procedure. Standard B-mode (2D) and M-mode images were taken in the short axis position at the level of the papillary muscles for each animal. Data analysis was performed by an experienced imager using the Visualsonics Vevo 770 cardiac analysis package. 2  M-modes were analyzed per animal with an average being taken for each.

### 2.3. Doppler Procedure

Aortic flow velocities were measured from the apical view using a 10 MHz pulsed Doppler probe and real time Doppler spectrum analyzer (Indus Instruments) [42]. Animals were taped to a temperature controlled board with electrocardiograph leads and anesthetized with 1% isoflurane gas in 100% oxygen by nose cone.

### 2.4. Statistical Analyses

Values are expressed as mean ± SEM or SD. Statistical differences were determined using Student's* t*-test or ANOVA followed by pairwise comparison using Tukey's test, Newman-Keuls or Dunnet's test as indicated. Significance was set at *P* < 0.05.

## 3. Results 

In these studies, we addressed if* in vivo* pretreatment, with the SP specific, NK1 receptor antagonist, aprepitant, or with the Rho-A inhibitor, fasudil, will serve as novel treatment options for viral-myocarditis.

Pretreatment with aprepitant an NK1R antagonist significantly inhibited all EMCV-induced manifestations such as mortality, increase in the heart-to-body weight ratio, cardiomyocyte hypertrophy, cardiac viral levels, and heart functional changes. When aprepitant was given after virus exposure, manifestations such as mortality, heart-to-body weight ratio increments, cardiomyocyte hypertrophy, and cardiac viral levels were significantly improved, but aprepitant posttreatment did not dramatically impact heart function. We demonstrated that although only posttreatment with fasudil reduced heart-to-body weight ratio, fasudil treatment on the whole (pre- or posttreatment) did not significantly impact mortality, cardiomyocyte hypertrophy, cardiac viral levels, or heart functional changes (Figure 7).

### 3.1. Effect of Aprepitant or Fasudil Treatment on EMCV-Induced Mortality

**Figure 1:**
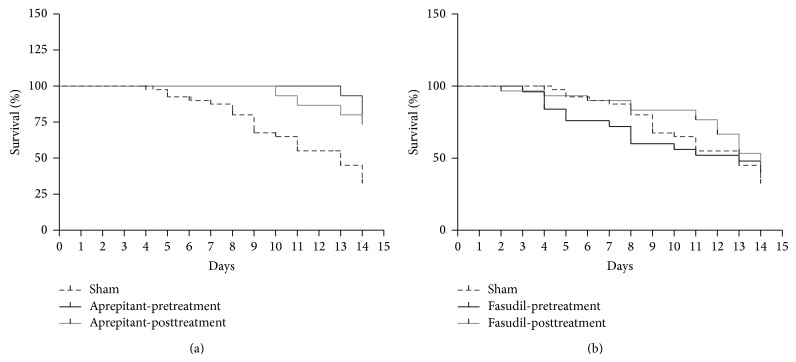
Effect of aprepitant or fasudil treatment on mortality rates in EMCV-infected mice. Effect of (a) aprepitant or (b) fasudil treatment on EMCV-induced mortality. Percent survival rate was determined in EMCV-infected mice with and without aprepitant or fasudil treatment over 14 days following infection (infected, with versus without aprepitant pretreatment, or infected, with versus without aprepitant posttreatment (both *P* < 0.05, Kaplan-Meier analysis), infected, with versus without fasudil pre- or posttreatment (both *P* > 0.05, Kaplan-Meier analysis) (*n* = 15–40)).

A mortality rate of 65 ± 7% was seen at day 14 in untreated wild type mice following infection with 50 pfu of the EMCV virus (*n* = 40; Figure 1(a)). Importantly, pretreatment as well as posttreatment with aprepitant in infected mice reduced mortality by 40% to 25 ± 7% (for both pre- and posttreatment) (*n* = 15 (both pre- and postaprepitant treatment groups) and *P* < 0.05, for both, Kaplan-Meier analysis, Figure 1(a)).

There was no significant difference between the mortality rate in the sham treated mice and the fasudil pre- or posttreated mice (*n* = 25 (fasudil pretreated) and *n* = 30 (fasudil posttreated); *P* > 0.05 for both, Kaplan-Meier analysis; Figure 1(b)).

### 3.2. Effect of Aprepitant or Fasudil Treatment on EMCV-Induced Heart Enlargement

**Figure 2:**
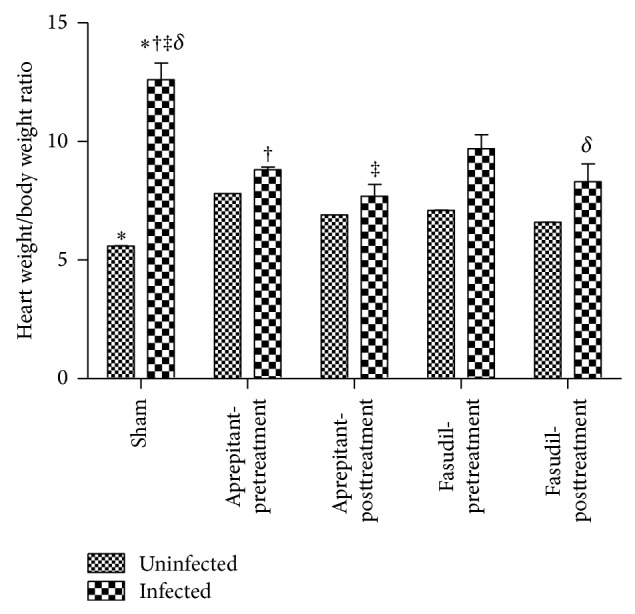
Effect of aprepitant or fasudil treatment on heart size in viral-myocarditis. Heart-to-body weight ratios of EMCV uninfected mice and infected mice, with and without aprepitant or fasudil treatment. Results are expressed as the mean ± SD of 2 separate experiments (Heart-to-body weight ratios (expressed in terms of milligrams of heart weight to milligrams of total body weight), of, ∗, uninfected versus infected, †, infected, with versus without aprepitant pretreatment, ‡, infected, with versus without aprepitant posttreatment and, *δ*, infected, with versus without fasudil posttreatment, mice (all *P* < 0.05, ANOVA) (*n* = 4–10)).

The mean heart-to-body weight ratio (expressed in terms of mg/g) following infection of 50 pfu of EMCV in mice was significantly increased in response to EMCV infection (12.6 ± 0.7 (*n* = 8) versus only 5.6 ± 0.76 (*n* = 5) in the control uninfected mice, *P* < 0.05, ANOVA and Tukey's post hoc test). Importantly, pretreatment with aprepitant in infected mice reduced heart-to-body weight ratio by 30% (8 ± 1 (*n* = 6) versus 12.6 ± 0.7 (*n* = 8), *P* < 0.05, ANOVA and Tukey's post hoc test; Figure 2) and posttreatment with aprepitant in infected mice reduced heart-to-body weight ratio by 39% (7.7 ± 0.5 (*n* = 6) versus 12.6 ± 0.7 (*n* = 8), *P* < 0.05, ANOVA and Tukey's post hoc test; Figure 2), to values not significantly different from uninfected control mice.

EMCV-induced change in heart-to-body weight ratio was not significantly impacted with fasudil pretreatment (9.8 ± 0.5 (*n* = 8) versus 12.6 ± 0.7 (*n* = 8); *P* > 0.05, ANOVA and Tukey's post hoc test; Figure 2). In contrast, posttreatment with fasudil in infected mice significantly reduced heart-to-body weight ratio by 34% (8 ± 0.7 (*n* = 10) versus 12.6 ± 0.7 (*n* = 8), *P* < 0.05, ANOVA and Tukey's post hoc test; Figure 2).

### 3.3. Effect of Aprepitant or Fasudil Treatment on EMCV-Induced Cardiac Inflammation

**Figure 3:**
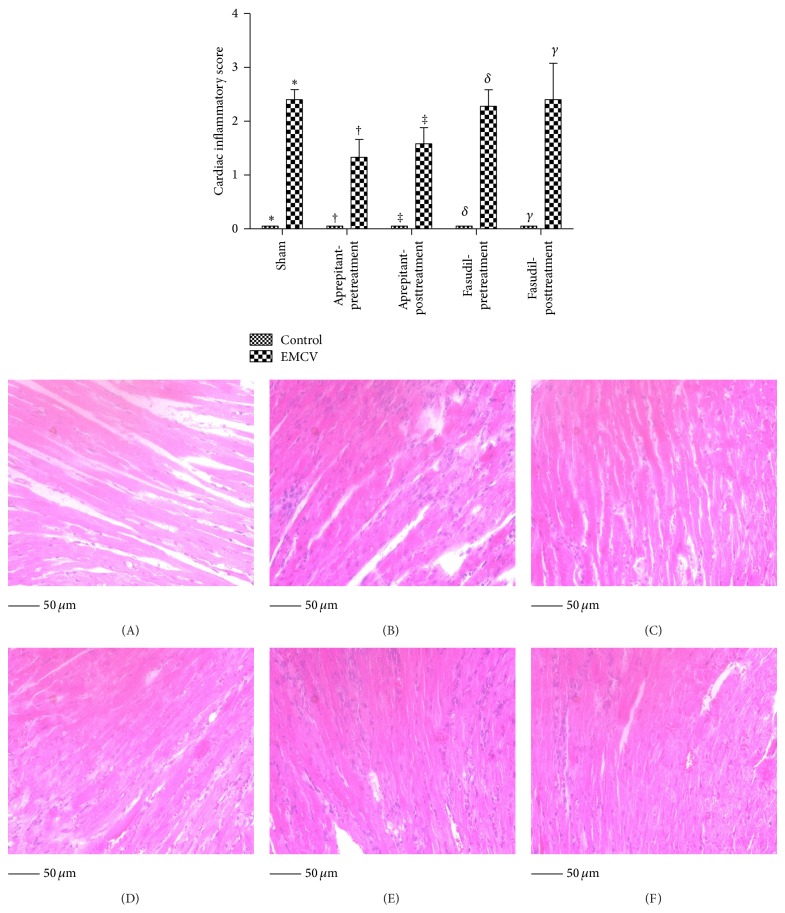
Effect of aprepitant or fasudil treatment on cardiac inflammation in viral-myocarditis. Paraffin-embedded heart sections were deparaffinized and stained with hematoxylin and eosin and were graded for cardiac inflammation. Cardiac inflammatory score of hearts derived from EMCV uninfected mice and infected mice, with and without aprepitant or fasudil treatment. Results are expressed as the mean ± SEM of 2 separate experiments (cardiac inflammatory score of, ∗, uninfected versus infected, †, uninfected versus infected, with aprepitant pretreatment, ‡, uninfected versus infected, with aprepitant posttreatment, *δ*, uninfected versus infected, with fasudil pretreatment and, *γ*, uninfected versus infected, with fasudil posttreatment, (*P* < 0.05 for all ANOVA and Tukey's post hoc test). Infected, with versus without pre- and postaprepitant treatment, or infected, with versus without fasudil pre- and posttreatment (all *P* > 0.05, ANOVA) (*n* = 4–7)). A representative H&E stained section from (a); (A) uninfected mouse, (B) EMCV-infected mouse, (C) EMCV-infected mouse with aprepitant pretreatment, (D) EMCV-infected mouse with aprepitant posttreatment, (E) EMCV-infected mouse with fasudil pretreatment, and (F) EMCV-infected mouse with fasudil posttreatment, depicting that EMCV-induced cardiac inflammation was not significantly impacted in aprepitant or fasudil treated mice.

The mean inflammatory score following infection of 50 pfu of EMCV in mice was significantly increased in response to EMCV infection (2.4 ± 0.2 (*n* = 5) versus only 0 ± 0 (*n* = 5) in the control uninfected mice (*P* < 0.05, ANOVA and Tukey's post hoc test)). Although the mean inflammatory score in infected mice with aprepitant pre- or posttreatment was lower than in infected mice without treatment, the differences were not statistically significant; aprepitant pretreatment (1.3 ± 0.3 (*n* = 4) versus 2.4 ± 0.2 (*n* = 5); *P* > 0.05, ANOVA and Tukey's post hoc test; Figure 3) or aprepitant posttreatment (1.6 ± 0.3 (*n* = 5) versus 2.4 ± 0.2 (*n* = 5); *P* > 0.05, Tukey's post hoc test; Figure 3).

Similarly, EMCV-induced change in cardiac inflammatory score was not significantly impacted with fasudil pretreatment (2.3 ± 0.3 (*n* = 7) versus 2.4 ± 0.2 (*n* = 5); *P* > 0.05, ANOVA and Tukey's post hoc test; Figure 3) or fasudil posttreatment (2.4 ± 0.7 (*n* = 5) versus 2.4 ± 0.2 (*n* = 5); *P* > 0.05, Tukey's post hoc test; Figure 3).

### 3.4. Effect of Aprepitant or Fasudil Treatment on EMCV-Induced Cardiomyocyte Hypertrophy

**Figure 4:**
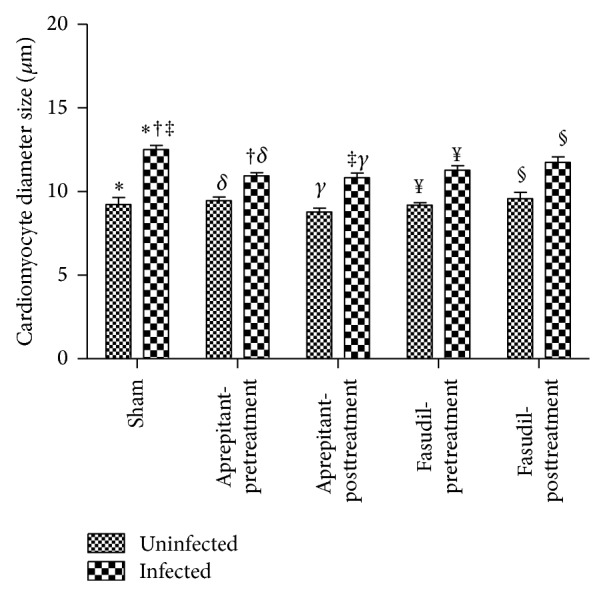
Effect of aprepitant or fasudil treatment on cardiomyocyte diameter in viral-myocarditis. Cardiomyocyte diameter (*μ*m) at the nucleus was determined in hearts of EMCV uninfected mice and infected mice, with and without aprepitant or fasudil treatment. Results are expressed as mean ± SEM (cardiomyocyte diameter of, ∗, uninfected versus infected, †, infected, with versus without aprepitant pretreatment, ‡, infected, with versus without aprepitant posttreatment, *δ*, uninfected versus infected, with aprepitant pretreatment, *γ*, uninfected versus infected, with aprepitant posttreatment, ¥, uninfected versus infected, with fasudil pretreatment and, §, uninfected versus infected, with fasudil posttreatment, (*P* < 0.05 for all ANOVA) (*n* = 4–9)).

The mean cardiomyocyte diameter following infection of EMCV in mice was significantly increased in response to EMCV infection (12.5 ± 0.3 *μ*m (*n* = 7) versus 9.2 ± 0.4 *μ*m (*n* = 5) in the control uninfected mice (*P* < 0.05, ANOVA and Tukey's post hoc test)). Importantly, pretreatment with aprepitant in infected mice reduced cardiomyocyte diameter by 12% (11 ± 0.2 *μ*m (*n* = 6) versus 12.5 ± 0.3 *μ*m (*n* = 7), *P* < 0.05, ANOVA and Tukey's post hoc test; Figure 4) and posttreatment with aprepitant in infected mice reduced cardiomyocyte diameter by 13% (11 ± 0.3 *μ*m (*n* = 6) versus 12.5 ± 0.3 *μ*m (*n* = 7), *P* < 0.05, ANOVA and Tukey's post hoc test; Figure 4), to values not significantly different from uninfected control mice.

EMCV-induced change in cardiomyocyte diameter was not significantly impacted with fasudil pretreatment (11.3 ± 0.3 (*n* = 8) versus 12.5 ± 0.3 *μ*m (*n* = 7); *P* > 0.05, ANOVA and Tukey's post hoc test; Figure 4) or fasudil posttreatment (11.7 ± 0.3 (*n* = 9) versus 12.5 ± 0.3 *μ*m (*n* = 7); *P* > 0.05, Tukey's post hoc test; Figure 4).

### 3.5. Effect of Aprepitant or Fasudil Treatment on Cardiac EMCV Viral Load

Viral load was significantly reduced in aprepitant pretreated and posttreated mice, whereas viral load was not significantly impacted with fasudil pre- or posttreatment.

**Figure 5:**
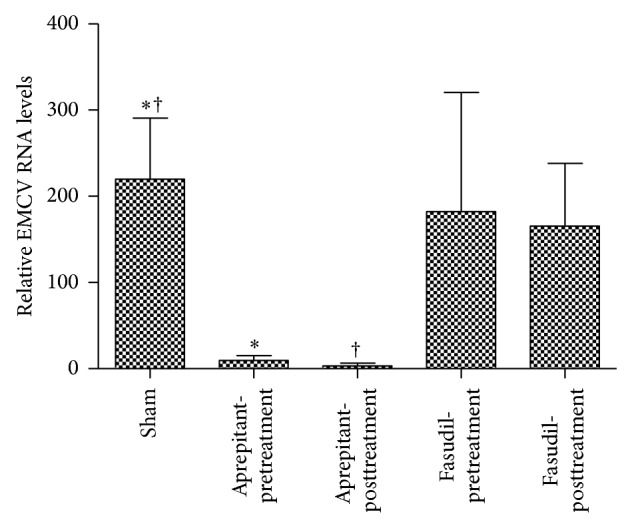
Effect of aprepitant or fasudil treatment on cardiac viral levels in viral-myocarditis. Relative cardiac EMCV RNA levels in EMCV-infected mice, with and without aprepitant or fasudil treatment. Results are expressed as the mean ± SEM (relative EMCV RNA levels of, ∗, infected, with versus without aprepitant pretreatment, †, infected, with versus without aprepitant posttreatment, (*P* < 0.05, Student's unpaired *t*-test) (*n* = 3-4)).

The relative EMCV RNA levels in mice without treatment 14 days after infection was 220 ± 71 (*n* = 3) in the WT mice. Importantly, pretreatment with aprepitant in infected mice reduced viral levels by 96% (9.6 ± 5.5 (*n* = 4), *P* < 0.05, Student's unpaired* t*-test; Figure 5) and posttreatment with aprepitant in infected mice reduced viral levels by 98% (3.2 ± 3.2 (*n* = 3) versus 220 ± 71 (*n* = 3), *P* < 0.05, Student's unpaired* t*-test; Figure 5).

There was no difference between the relative EMCV RNA levels in mice with and without fasudil pretreatment (182 ± 138 (*n* = 3) versus 220 ± 71 (*n* = 3); *P* > 0.05, Student's unpaired* t*-test; Figure 5 or fasudil posttreatment) (165 ± 73 (*n* = 3) versus 220 ± 71 (*n* = 3); *P* > 0.05, Student's unpaired* t*-test; Figure 5).

### 3.6. Effect of Aprepitant or Fasudil Treatment on EMCV-Induced Heart Functions

**Figure 6:**
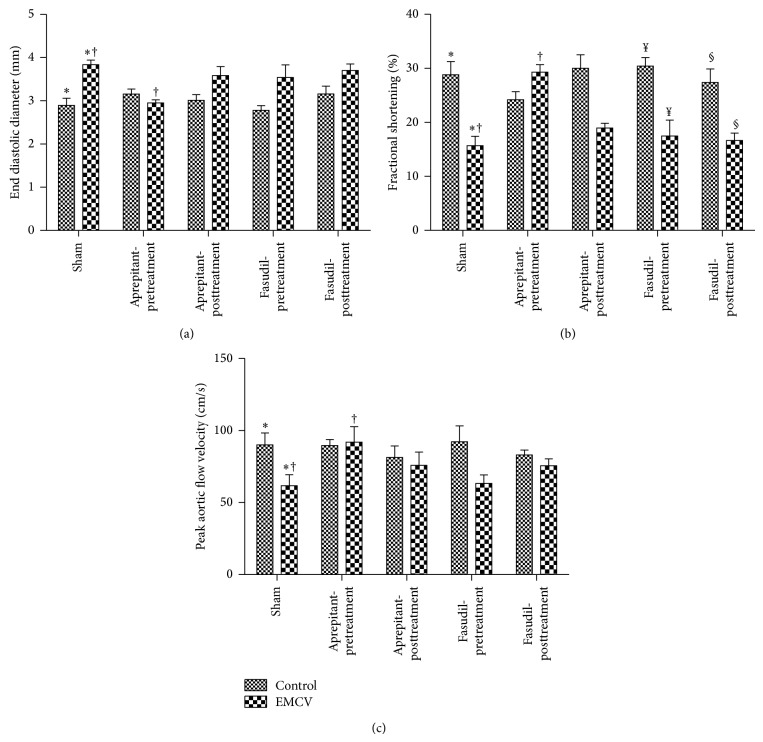
Effect of aprepitant or fasudil treatment on heart functional changes in viral-myocarditis. (a) End systolic diameter (ESD), (b) fractional shortening, and (c) peak aortic flow velocity were determined by ECHO and Doppler in hearts of EMCV uninfected mice and infected mice, with and without aprepitant or fasudil treatment. Results are expressed as the mean ± SEM (∗, uninfected versus infected, †, infected, with versus without aprepitant pretreatment, ¥, uninfected versus infected, with fasudil pretreatment and, §, uninfected versus infected, with fasudil posttreatment, (*P* < 0.05 for all ANOVA) (*n* = 4–8)).

**Figure 7:**
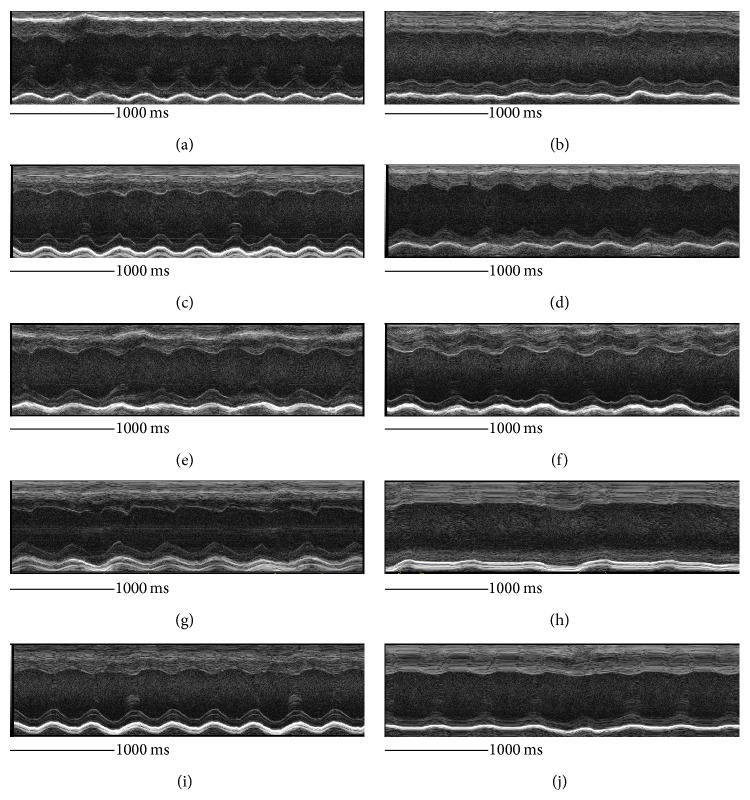
ECHO cardiographic representation of effects of aprepitant or fasudil treatment in viral-myocarditis. A representative ECHO reading from (a) uninfected mouse, (b) EMCV-infected mouse, (c) uninfected mouse with aprepitant pretreatment, (d) EMCV-infected mouse with aprepitant pretreatment, (e) uninfected mouse with aprepitant posttreatment, (f) EMCV-infected mouse with aprepitant posttreatment, (g) uninfected mouse with fasudil pretreatment, (h) EMCV-infected mouse with fasudil pretreatment, (i) uninfected mouse with fasudil posttreatment, and (j) EMCV-infected mouse with fasudil posttreatment.

End systolic diameter (ESD) was significantly increased in response to EMCV infection. The mean ESD following infection in mice was increased 31%, 3.8 ± 0.1 mm (*n* = 8) versus only 2.9 ± 0.2 mm (*n* = 5), in the control uninfected mice (*P* < 0.05, ANOVA and Newman-Keul's post hoc test). Importantly, pretreatment with aprepitant in infected mice reduced the effects of ECMV on ESD by 24% (2.9 ± 0.07 mm (*n* = 4) versus 3.8 ± 0.1 mm (*n* = 8), *P* < 0.05, ANOVA and Newman-Keul's post hoc test; Figure 6(a)).

EMCV-induced change in ESD was not significantly impacted either with aprepitant posttreatment or with fasudil pre- as well as posttreatment. There was no difference between the mean ESD in infected mice with and without aprepitant posttreatment (3.6 ± 0.2 mm (*n* = 4) versus 3.8 ± 0.1 mm (*n* = 8); *P* > 0.05, ANOVA and Newman-Keul's post hoc test; Figure 6(a)). Similarly, there was no difference between the mean fractional shortening in infected mice with and without fasudil pretreatment (3.5 ± 0.3 mm (*n* = 6) versus 3.8 ± 0.1 mm (*n* = 8); *P* > 0.05, ANOVA and Newman-Keul's post hoc test; Figure 6(a)) or fasudil posttreatment (3.7 ± 0.1 mm (*n* = 7) versus 3.8 ± 0.1 mm (*n* = 8); *P* > 0.05, ANOVA and Newman-Keul's post hoc test; Figure 6(a)).

We also measured end diastolic diameter (EDD). EDD levels were not significantly increased in response to EMCV infection. Aprepitant or fasudil treatment did not significantly impact EDD. Although the mean EDD following infection in mice was increased 13%; 4.6 ± 0.1 mm (*n* = 8) versus only 4 ± 0.1 mm (*n* = 5), in the control uninfected mice, the differences were not statistically significant (*P* > 0.05, ANOVA and Newman-Keul's post hoc test). Similarly, although pretreatment with aprepitant in infected mice reduced the effects of ECMV on EDD by 8%, the differences were not statistically significant (4.2 ± 0.06 mm (*n* = 4) versus 4.6 ± 0.1 mm (*n* = 8), *P* > 0.05, ANOVA and Newman-Keul's post hoc test). Similarly, although posttreatment with aprepitant in infected mice reduced the effects of ECMV on EDD by 7% the differences were not statistically significant (4.2 ± 0.06 mm (*n* = 5) versus 4.6 ± 0.1 mm (*n* = 8); *P* > 0.05, ANOVA and Newman-Keul's post hoc test). Also, EDD was not significantly impacted with fasudil treatment. There was no difference between the mean EDD in infected mice with and without fasudil pretreatment (4.3 ± 0.2 mm (*n* = 6) versus 4.6 ± 0.1 mm (*n* = 8); *P* > 0.05, ANOVA and Newman-Keul's post hoc test) or fasudil posttreatment (4.5 ± 0.2 mm (*n* = 7) versus 4.6 ± 0.1 mm (*n* = 8); *P* > 0.05, ANOVA and Newman-Keul's post hoc test).

### 3.7. Fractional Shortening Was Significantly Decreased in response to EMCV Infection

The mean fractional shortening following infection in mice was only 16 ± 2% (*n* = 8) versus 29 ± 2% (*n* = 5), in the control uninfected mice (*P* < 0.05, ANOVA and Newman-Keul's post hoc test). Importantly, pretreatment with aprepitant in infected mice ablated any effect of the ECMV on fractional shortening (29 ± 1% (*n* = 4) versus 16 ± 2% (*n* = 8), *P* < 0.05, ANOVA and Newman-Keul's post hoc test; Figure 6(b)), to values similar to that seen in uninfected control mice.

EMCV-induced decrease in fractional shortening was not significantly impacted either with aprepitant posttreatment (19 ± 1% (*n* = 4)) or with fasudil pre- as well as posttreatment. There was no difference between the mean fractional shortening in infected mice with and without aprepitant posttreatment (19 ± 1% (*n* = 4) versus 16 ± 2% (*n* = 8); *P* > 0.05, ANOVA and Newman-Keul's post hoc test; Figure 6(b)). Similarly, there was no difference between the mean fractional shortening in infected mice with and without fasudil pretreatment (17 ± 3% (*n* = 6) versus 16 ± 5% (*n* = 8); *P* > 0.05, ANOVA and Newman-Keul's post hoc test; Figure 6(b)) or fasudil posttreatment 17 ± 1% (*n* = 7) versus 16 ± 5% (*n* = 8); *P* > 0.05, ANOVA and Newman-Keul's post hoc test; Figure 6(b).

Peak aortic flow velocity was significantly reduced in response to EMCV infection. The mean peak aortic flow velocity following infection in mice was only 62 ± 8 cm/sec (*n* = 7) versus 90 ± 8 cm/sec (*n* = 7) in the control uninfected mice (*P* < 0.05, ANOVA and Dunnett's post hoc test). Importantly, pretreatment with aprepitant in infected mice increased peak aortic flow velocity by 49% (92 ± 11 cm/sec (*n* = 5) versus 62 ± 8 cm/sec (*n* = 7), *P* < 0.05, ANOVA and Dunnett's post hoc test; Figure 6(c)), similar to that seen in uninfected control mice.

EMCV-induced change in peak aortic flow velocity was not significantly impacted either with aprepitant posttreatment or with fasudil pre- as well as posttreatment. There was no difference between the mean peak aortic velocity in infected mice with and without aprepitant posttreatment (76 ± 9 (*n* = 5) versus 62 ± 8 cm/sec (*n* = 7); *P* > 0.05, ANOVA and Dunnett's post hoc test; Figure 6(c)). Similarly, there was no difference between the mean peak aortic velocity in infected mice with and without fasudil pretreatment (63 ± 6 (*n* = 6) versus 62 ± 8 cm/sec (*n* = 7); *P* > 0.05, ANOVA and Dunnett's post hoc test; Figure 6(c)) or fasudil posttreatment (76 ± 5 (*n* = 7) versus 62 ± 8 cm/sec (*n* = 7); *P* > 0.05, ANOVA and Tukey's post hoc test; Figure 6(c)).

## 4. Discussion

Viral-myocarditis is an important cause of heart failure among adolescents and young adults [1]; currently, there is no specific treatment for this disease [2–4, 44]. Encephalomyocarditis virus (EMCV) infection causes a fatal disease in mice marked by dilated cardiomyopathy and congestive heart failure and is used as an experimental model to study viral-myocarditis [6–9]. Our earlier studies have shown that that SP is required for the pathogenesis of EMCV infection in mice [8]. The SP receptor, neurokinin 1 receptor (NK1R), is a G-protein coupled receptor. Binding of SP to NK1R leads to activation of upstream regulators of RhoA activity [23, 24]. Signals elicited by G-protein coupled receptor activation have shown to induce cardiomyocyte hypertrophy [25, 26]. Mice overexpressing RhoA develop a lethal, dilated cardiomyopathy and heart failure [34, 35].

In the current studies, we examined whether blocking of NK1R-signaling (with aprepitant) or RhoA activity* in vivo* with fasudil will improve survival and prevent manifestations of myocarditis (as studied by echo and Doppler).

We determined whether SP mediated pathogenesis of viral-myocarditis is via NK1R-signaling and downstream RhoA activation. We determined whether blocking of NK1R-signaling with aprepitant (NK1R antagonist) will improve survival by inhibiting manifestations of myocarditis. We determined that pretreatment with aprepitant reduced mortality, prevented the increase in heart-to-body weight ratio, minimized the increase in end systolic diameter (ESD), reduced the increase in cardiomyocyte diameter, and increased fractional shortening and peak aortic flow velocity compared to that of infected, saline treated mice. We also determined that although posttreatment of aprepitant was effective in inhibiting manifestations such as mortality and increases in cardiac viral load, heart-to-body weight ratio, and cardiomyocyte diameter, it did not significantly impact heart functional parameters. Although the current studies showed a significant inhibition of the pathogenesis of disease with NK1 receptor antagonist, both pretreatment and posttreatment did not completely prevent disease. This could be possible because SP binds with high affinity to NK1R. However, it can also bind to NK2R and NK3R, although with much lower affinity. Therefore, it may be possible that even in the absence of NK1R, SP may be mediating its effects through interaction with NK2R and NK3R. Therefore, we may not have observed complete ablation of pathogenesis in the NK1R treated mice. Currently, there are no antagonists that antagonize all 3 receptors at the same time; therefore we have to rely on studies using individual antagonists that antagonize each receptor. However, as mentioned in Section 1, we chose to study effects of NK1 antagonist, since that is the high affinity receptor and also because that is the predominant receptor that mediates SP responses in the heart [37]. Nevertheless, it will still be interesting to study the effect of NK2 and NK3 antagonists in myocarditis and, as part of our future goal, we plan to study the effect of the other 2 receptor antagonists.

Our previous results [8] wherein we have used SP precursor knockout mice demonstrated complete reversal of manifestations of murine viral-myocarditis, whereas our current studies using pharmacological inhibition showed reduction, although significant but not complete prevention of the manifestations. One of the reasons this could be possible could be due to the possibility of the additive effects of neurokinin A contributing to the manifestations of EMCV myocarditis. The SP precursor knockout mice are deficient in neurokinin A as well as SP, whereas the NK1R predominantly inhibits SP activity. In order to dissect out the effects of SP versus neurokinin A, as part of our future studies, we will study the effect of NK2 antagonists that is the predominant receptor that neurokinin A interacts with.

Importantly, we demonstrated that aprepitant treatment not only inhibited the manifestations of murine viral-myocarditis, but also decreased cardiac EMCV viral RNA levels compared to aprepitant untreated, EMCV-infected mice. We do not know the mechanisms which could have resulted in decreased viral load in the aprepitant treated mice. We anticipate that it could either be due to one or more of the following reasons; substance P may be directly responsible for enhancing viral replication, or it may be that aprepitant has direct antiviral effects, or it could be possible that SP and/or aprepitant indirectly via some unknown mechanism may be causing reduced ability of the virus to reach the heart from the peritoneal cavity where it was injected, and finally it may be possible that the NK1R may be important for viral entry. We anticipate the possibility that SP may directly enhance viral replication and the possibility that aprepitant may serve as an antiviral agent to be very relevant based on other studies outlined below wherein SP has shown to enhance viral replication and aprepitant has demonstrated antiviral capabilities. Substance P has shown to enhance HIV-1 replication in latently infected human immune cells such as a latently infected promonocytic cell line (U1) and a T lymphocyte line (ACH-2) [45]. Furthermore, the addition of SP to the cultures of latently infected peripheral blood mononuclear cells isolated from HIV-1-infected patients has shown to enhance HIV-1* gag* gene expression [45]. In contrast, aprepitant has shown to inhibit drug-resistant HIV-1 infection of macrophages* in vitro* [46]. Also when aprepitant was added to microglia cultures infected with CSF-derived HIV-1 strains (JAGO or JRFL), it led to significantly inhibited viral replication. These studies implicate that the decreased viral levels we demonstrated in the heart of NK1R antagonist, aprepitant treated mice could be due to inhibition of viral enhancing effects of SP and/or due to direct antiviral effects of aprepitant. Further studies in our laboratory will be aimed at determining the direct role of SP and aprepitant, on EMCV viral replication and viral migration to the heart.

The current studies demonstrate that aprepitant is effective as a preventive measure; aprepitant treatment before viral infection inhibits all manifestations of EMCV infection, whilst administration of aprepitant 5 days after infection, although effective in inhibiting manifestations such as mortality and increases in cardiac viral load, heart-to-body weight ratio, and cardiomyocyte diameter, it did not significantly impact heart functional parameters. We do not know the exact reason why heart functional parameters were not improved in spite of other manifestations showing an improvement in the mice that were administered aprepitant after infection; possible reasons could include the following: it could be possible that there may be an underlying effect that occurs in the first few days of infection that despite subsequent depletion of the virus may be already in effect in the postaprepitant treated group. Dehlin and Levick [47] have recently shown that NK-1R blockade prevented fibrosis in the hypertensive heart; visual examination of heart sections from aprepitant pretreated mice in our study also showed reduced fibrosis compared to untreated or aprepitant posttreated mice (results not shown). Therefore underlying effects such as fibrosis that may occur in the first few days of infection despite subsequent depletion of the virus may be already in effect in the postaprepitant treated group and could explain why heart functional parameters were not improved in spite of the fact that other manifestations including viral levels showed an improvement in the mice that were administered aprepitant after infection. Another reason could be that the timing of aprepitant after infection may have been well into initiation of the functional manifestations and we may have been able to prevent the heart functional changes if we had administered the drug on day 3 after infection instead of day 5 after infection. Alternatively and/or additionally, it may be possible that a lower dosage of aprepitant is sufficient to prevent all manifestations of disease, while a higher dose of aprepitant is needed to exert complete therapeutic effects. In order to address these 2 possibilities, as part of our future studies, we plan to assess functional parameters in mice that are administered aprepitant at earlier times after infection. In addition, as part of our future studies, we plan to perform the studies using higher doses of aprepitant before we definitively conclude that aprepitant administration after infection is only partially effective. Similar studies will be conducted with fasudil before we definitely conclude that fasudil does not have any effect as a preventive or therapeutic drug. An important point to be noted is that the doses of aprepitant and fasudil that we have used for the current study do not have any detrimental effect by themselves on cardiac functions. We determined that none of the cardiac parameters that we have studied including mortality, heart-to-body weight ratio, cardiomyocyte diameter, and heart functional parameters were significantly different between the sham treated uninfected mice versus that of the aprepitant or fasudil treated uninfected mice.

A question also arises as to the reason why posttreatment of aprepitant was beneficial as demonstrated by decrease in mortality in spite of decreased heart functions. Possible explanations could include that in the EMCV model of murine infection, dilated cardiomyopathy does not set in until later in infection, possibly beginning at days 10–12 after infection. Therefore if we terminate the experiment at a later time-point than 14 days, we may observe increased mortality in the aprepitant posttreated mice specifically due to declining heart functions. Alternatively, we may determine that in the aprepitant posttreated mice, the compensatory phase leading to decreased heart functions may rectify itself leading to survival as a result of improving heart functions. In order to address if the former scenario is the explanation for lack of association between survival rate and declining heart functions in the aprepitant posttreated group, as part of our future studies, we will study the survival rate and heart functions in mice at later times following infection and also compare if there is an improvement with changes in dosage and administration regime.

In the current studies, we also determined if SP mediated pathogenesis is mediated via downstream RhoA activation. We anticipated that if SP/NK1R mediated pathogenesis of myocarditis is mediated via RhoA-signaling, RhoA levels will be elevated in EMCV-infected mice and RhoA inhibitors will inhibit pathogenesis of EMCV infection. We determined that RhoA levels were significantly increased in response to EMCV infection compared to uninfected mice (results not shown). Furthermore, although heart enlargement in response to EMCV infection was significantly reduced only in fasudil posttreated mice, none of the other manifestations were improved in either the fasudil pre- or posttreated mice. The predominant inability of fasudil to prevent or treat manifestations of disease may be because there may be multiple signaling mechanisms, such as other GTPases involved in SP mediated responses. Therefore blocking RhoA alone may not have resulted in inhibition of the SP induced responses. As part of our future studies, we will look for the role in pathogenesis of other GTPases like Rac and Cdc 42 that are known to be activated by SP [48]. Other studies have shown that NK1R activation leads to activation of upstream regulators of RhoA activity [23, 24]. Therefore, we have no reason to doubt that in our system NK1R activation does not lead to Rho-A activation or that NK1R antagonism will not lead to inhibition of Rho-A activation. The inability of fasudil to reverse pathogenesis of EMCV infection could imply that although NK-1R may be regulating Rho-A, it may mean that the Rho-A pathway may mediate other effects not related to the effects measured in this paper.

The role of SP and NK1R in other experimental models of cardiac-remodeling has been addressed by few investigators [47, 49–51]. For example, using a model of chronic volume-overload induced heart failure; Meléndez et al. demonstrated that mice that have a deletion of the SP gene were protected from developing left ventricular hypertrophy and increases in right ventricular mass, indicating protection from heart failure [49]. While other studies have demonstrated that SP was increased in the cardiac lesions of magnesium-deficient mice and that blockade of NK1R improved diastolic and systolic function and improved ischemia/reperfusion related manifestations [50, 51]. The above-mentioned studies studying the role of NK1R antagonist on pathogenesis induced as a result of a magnesium-deficient diet use NK1R antagonist as a preventive regime. This study administers NK1R antagonist starting the same time as the magnesium-deficient diet and hence coincides with our studies wherein we also show that a preventive regime of NK1R antagonist improved pathogenesis in the context of EMCV infection.

In conclusion, these findings indicate that SP contributes to cardiac-remodeling and dysfunction following ECMV infection via its high affinity receptor, NK1R. However SP/NK1R mediated pathogenesis of EMCV-induced myocarditis is not significantly mediated via RhoA-signaling. These studies implicate that pharmacological inhibition of NK1 receptor may serve as novel treatment option for viral-myocarditis.
